# Nutrient structure dynamics and microbial communities at the water–sediment interface in an extremely acidic lake in northern Patagonia

**DOI:** 10.3389/fmicb.2024.1335978

**Published:** 2024-02-12

**Authors:** Mayra Cuevas, Issotta Francisco, Fernando Díaz-González, Mónica Diaz, Raquel Quatrini, Guadalupe Beamud, Fernando Pedrozo, Pedro Temporetti

**Affiliations:** ^1^Instituto de Investigaciones en Biodiversidad y Medioambiente (INIBIOMA), Centro Regional Universitario Bariloche-UNComahue, CCT-Patagonia Norte, CONICET, San Carlos de Bariloche, Argentina; ^2^Centro Científico y Tecnológico de Excelencia Ciencia & Vida, Fundación Ciencia & Vida, Santiago, Chile; ^3^Department of Molecular Genetics and Microbiology, School of Biological Sciences, P. Universidad Católica de Chile, Santiago, Chile; ^4^Facultad de Medicina y Ciencia, Universidad San Sebastián, Santiago, Chile

**Keywords:** bacteria, nutrients, microbial communities, water–sediment interface, acidic lake, Argentinean Patagonia

## Abstract

Lake Caviahue (37° 50 ‘S and 71° 06’ W; Patagonia, Argentina) is an extreme case of a glacial, naturally acidic, aquatic environment (pH ~ 3). Knowledge of the bacterial communities in the water column of this lake, is incipient, with a basal quantification of the bacterioplankton abundance distribution in the North and South Basins of Lake Caviahue, and the described the presence of sulfur and iron oxidizing bacteria in the lake sediments. The role that bacterioplankton plays in nutrient utilization and recycling in this environment, especially in the phosphorus cycle, has not been studied. In this work, we explore this aspect in further depth by assessing the diversity of pelagic, littoral and sediment bacteria, using state of the art molecular methods and identifying the differences and commonalties in the composition of the cognate communities. Also, we investigate the interactions between the sediments of Lake Caviahue and the microbial communities present in both sediments, pore water and the water column, to comprehend the ecological relationships driving nutrient structure and fluxes, with a special focus on carbon, nitrogen, and phosphorus. Two major environmental patterns were observed: (a) one distinguishing the surface water samples due to temperature, Fe^2+^, and electrical conductivity, and (b) another distinguishing winter and summer samples due to the high pH and increasing concentrations of N-NH_4_^+^, DOC and SO_4_^2−^, from autumn and spring samples with high soluble reactive phosphorus (SRP) and iron concentrations. The largest bacterial abundance was found in autumn, alongside higher levels of dissolved phosphorus, iron forms, and increased conductivity. The highest values of bacterial biomass were found in the bottom strata of the lake, which is also where the greatest diversity in microbial communities was found. The experiments using continuous flow column microcosms showed that microbial growth over time, in both the test and control columns, was accompanied by a decrease in the concentration of dissolved nutrients (SRP and N-NH_4_^+^), providing proof that sediment microorganisms are active and contribute significantly to nutrient utilization/mobilization.

## Introduction

The role of the bacterial communities in the biochemical transformation of organic matter, both particulate and dissolved, is fundamental to the nutrient structure dynamics, and energy flow in aquatic systems (Wetzel, 2001; Song et al., 2011; Dai et al., 2016). These communities are also key in the biogeochemical cycling of elements that serve as nutrients for microorganisms at the base of the food web (Logue and Lindström, 2008) and compete for inorganic and organic nutrients with algae (Kamjunke et al., 2008).

In freshwater lakes, sediments and their associated pore water interact closely. In this sense, there is a constant transfer of bacteria between habitats with the consequent alteration of the cognate microbial communities, as reflected by changes in the patterns of bacterial occurrence and diversity (Keshri et al., 2017). According to Bloesch (2009), the sediment–water interface is a zone of intensive decomposition of organic matter caused by bacteria. Sediment, especially in the pore water and near the sediment–water interface, plays an important role in removing and precipitating chemical elements from surface water and/or releasing them into the water column (Donahoe and Liu, 1998; Cook et al., 2018). In this way, the nutrient fluxes at the water–sediment interface are not only influenced by the nutrient concentration gradient between these compartments, but also by the content and composition of organic matter and the activity of bacteria, among others (Song et al., 2011; Zhang et al., 2013, 2018).

According to Keshri et al. (2017), sediments are characterized by high microbial biomass and taxon richness compared to the upper water column. These receive a high deposition of microbes and organic matter from the upper water layer and provide a matrix of complex nutrients and solid surfaces for microorganism’s growth. In this way, sediment bacteria play a vital role in the degradation and transformation of organic matter (Keshri et al., 2017) and nutrients (e.g., dissolved organic carbon, nitrogen, and phosphorus) (Dai et al., 2016; Zhang et al., 2018). Changes in the composition of microbial communities can significantly impact the biogeochemical environments of sediments (Song et al., 2011). Experiments performed by Gachter et al. (1988) suggested that sediment’s bacteria can rapidly take up and release soluble reactive phosphorus (SRP), depending on redox conditions, but the sterilization of oxic sediments decreased their SRP sorption capacity. Likewise, Song et al. (2011) concluded that the total phosphorus (TP) concentration of sediments from Lake Dongping (China) was determinant for the sediment bacterial community structure. Also, given that the heterotrophic bacterioplankton and the autotrophic phytoplankton in the water column of a water-body may be limited by different nutrients, an increase in the nutrient load may have different effects on bacterial and algal biomass and diversity (Danger et al., 2007; Xu et al., 2014). Consequently, investigating the composition of the bacterial community is indispensable to properly understand microbial processes and mater cycling in lakes (Keshri et al., 2017).

Several studies have focused on understanding the trophic status and microbial communities’ composition and variations in acidic environments generated by acid mine drainage (Löhr et al., 2006; He et al., 2019; Lukhele et al., 2019) or caused by sulfide/sulfate weathering in brine lakes (Escudero et al., 2018; Zaikova et al., 2018; Sánchez-España et al., 2020). The studies on the microbiota of sediments from acidic environments are yet more scant (Wassel and Mills, 1983; Rao et al., 1984; Falagán et al., 2015; Zhang et al., 2018; Aerts et al., 2019; Willis et al., 2019). Even if similarities and differences of bacterial richness and diversity in water columns in acidic environments is beginning to emerge (García-Moyano et al., 2012; Hou et al., 2019; Arce-Rodríguez et al., 2020; Grettenberger et al., 2020), our current understanding of the types of microorganisms and the roles exerted by them in sediments from natural volcanic acidic lakes in comparison with anthropogenic acidic lakes is very limited (e.g., Rincón-Molina et al., 2019).

Among acidic volcanic lakes in the word, Lake Caviahue (Patagonia, Argentina) has a number of particularities that differentiate it from other basins studied thus far: it has developed entirely in a volcanic rock environment, and the lake lodges in a volcanic depression (Delpino et al., 1997) eroded by glacial action (Pesce, 1989). The water chemistry has been extensively studied (Pedrozo et al., 2001; Gammons et al., 2005; Geller et al., 2006; Parker et al., 2008; Varekamp, 2008; Varekamp et al., 2009). Phytoplankton and zooplankton diversity are low in this environment (Pedrozo et al., 2001), with Chlorophyta Trebouxiophyceae *Keratococcus rhaphidioides* as dominant species (>90% of the total abundance) (Beamud et al., 2007). A few other species (*Pseudococcomyxa simplex*, *Watanabea* sp., *Chlamydomonas acidophila, Ochromonas* sp., and *Palmellopsis* sp.) have also been found in the lake in low numbers. The only representative of the zooplankton community is a species of Bdelloideo rotifer, *Philodina* sp. The trophic web in Lake Caviahue is simple: there are no copepods or crustaceans, and no fish (Beamud et al., 2010). Understanding of the diversity and ecological roles of microbial communities within the water column of Lake Caviahue is still incipient, with reported basal quantifications of the bacterioplankton abundance and distribution in the North and South Basin of the lake, and the described the presence of sulfur and iron oxidizing bacteria in the lake sediments (Pedrozo et al., 2001; Wendt-Potthoff and Koschorreck, 2002).

The aim of this work was to study the role that bacterioplankton plays in nutrient utilization and recycling, especially in the phosphorus cycle, in acidic Lake Caviahue. Specifically, the use of nutrients in interstitial water and at the water–sediment interface by sediment bacteria was studied to further understand the nutrient dynamics in natural acidic lakes. On the other hand, we have assessed the diversity of pelagic, littoral and sediment microbial communities using state of the art molecular methods, identifying the variations and ecological relationships of the communities of each strata.

## Materials and methods

### Study area

**Figure 1:**
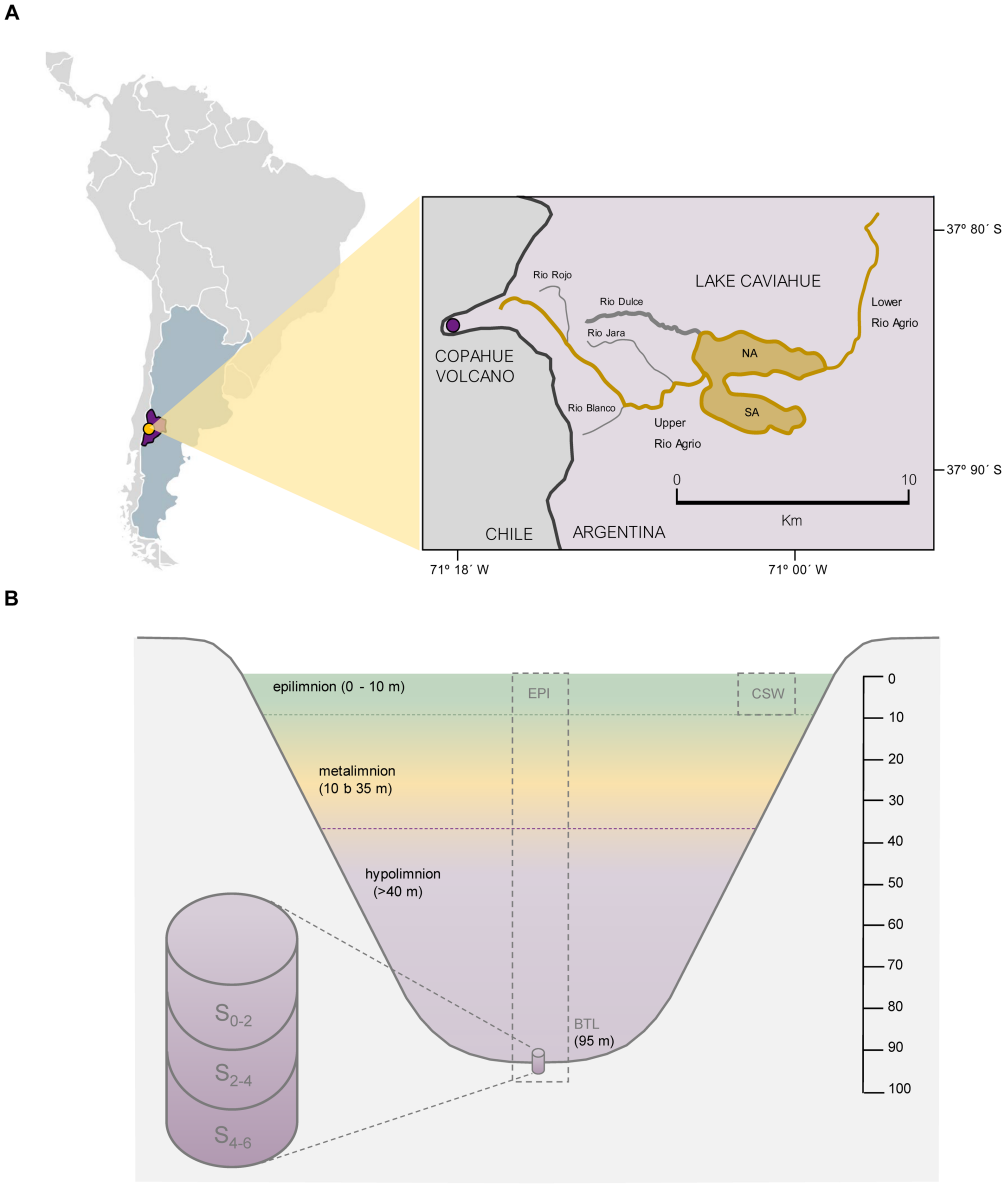
Study area and Lake Caviahue characteristics. **(A)** Map of the study area at Neuquén province, Argentina. Sampling at Lake Caviahue (LC) at deepest depth in the North (NA, 95 m) and South (SA, 65 m) arms. **(B)** Profile of Lake Caviahue’s northern arm at its point of maximum depth. Sampling points: CSW: costal shallow water, EPI: epilimnion (at 5 m), metalimnion (at 20 m), and bottom sediments samples: 0–2 cm, 2–4 cm, and 4–6 cm layers. Dashed line (purple): location of the sampling sites in the NA and SA.

Lake Caviahue (Figure 1A) is a large lake carved by glaciers. It is located inside the Caviahue caldera at 1,600 meter above sea level (m.a.s.l), within the Copahue-Caviahue Provincial Park (37° 50 ‘S and 71° 06’ W). It is an extremely acidic lake (pH 2.0–3.0) (Pedrozo et al., 2001, 2008a) due to the influence of the Upper Agrio river (UA). This river is born at 2740 m.a.s.l. on the eastern slope of the Copahue Volcano (Figure 1), and is located approximately 200 m below the crater rim (Agusto and Varekamp, 2016). The UA (1.10 m^3^ s^−1^) forms a delta as it empties into Lake Caviahue, providing water with pH between 0.78–3.50 (20-year range). The lake, with an area of 9.2 km^2^, has a horseshoe shape open to the east, presenting two arms: North Arm (NA; maximum depth of 95 m) and South Arm (SA; maximum depth of 72 m). Water residence time varies between 2.6 (Rapacioli, 1985) to 3.5 (Varekamp, 2003, 2008) years. The lake stratifies thermally between January and March (thermocline between 20 and 40 m depth), and during the rest of the year it remains mixed or with a very low temperature gradient (Beamud et al., 2007).

### Sample collection and field procedures

Samples were collected from pelagic and littoral waters and sediments of the NA of Lake Caviahue (Figure 1A). The NA littoral shallow waters samples (CSW) were obtained from the water column in February 2019. A total volume of 25 L of water was collected in plastic drums, disinfected with 70% ethanol, and rinsed with water from the collection point (37.86522 S 71.03700 W), with the help of a transfer pump. The sample was decanted and prefiltered through sterile 3 mm Whatman filters to remove particulate matter, and then filtered through 0.22 μm pore sizes filters with a 500 mL Nalgene vacuum filtration system. The saturated filters were stored at −20°C during the field campaign, and then kept at −80°C until processing.

At the deepest point of NA (95 m) and SA (72 m), water column samples for chemical analysis and bacteria abundance were taken from three depths: surface (EPI, epilimnion, 5 m), middle (MET, metalimnion, 20 m) and bottom layer (BTL) at the maximum depth (Figure 1B). Sampling was performed once per season (summer, autumn, winter and spring), throughout the years 2016–2017. In this work, the bottom water sample corresponds to the water–sediment interface recovered from the first 10 cm of water above the sediments surface. This zone is the one with the greatest interaction in nutrient exchange between sediments and the water column (Golterman, 2004). Water samples were taken with a Van Dorn bottle (3.5 L capacity PVC Vertical Water Bottle). The samples were stored in plastic containers under cold and dark conditions (APHA, 2017), until further analysis in the laboratory. Bottom samples were collected by siphoning the water of the first centimeters (5 cm) above the sediment core which was obtained using a UWITEC (USC 06000) sampler with an acrylic tube of 6 cm in diameter and 60 cm in length.

Water column temperature, pH, conductivity, and oxygen were measured *in situ* with a multiparameter probe (YSI 6600 V2 or Hanna HI9829). Transparency was measured with a Secchi disk. In water-column samples, total phosphorus, and nitrogen (TP and TN, respectively) were determined on the unfiltered fraction, while dissolved phosphorus (SRP) and ammonium (N-NH_4_^+^) on the filtered fraction (0.45 μm pore). All chemical analyses were carried out by spectrophotometry (Metrolab 1,100 spectrophotometer): nutrients (TP, SRP, and N-NH_4_^+^) according to recommendations to APHA (2017); Golterman et al. (1978) and Grasshoff et al. (1983); sulfates (SO_4_^2−^) were determined by turbidimetry (Method Hach 8,051); total iron (TFe), ferrous ion (Fe^2+^), and aluminum (Al^3+^) were determined according to Hach methods (N^o^: 8008, 8,146 and 8,012, respectively). Ferric ion (Fe^3+^) was calculated as the difference between TFe and Fe^2+^. Dissolved Organic Carbon (DOC) was determined on a filtered sample (0.22 μm pore glass filter, previously muffled) with a total C analyzer at the Institute of Theoretical and Applied Physicochemical Research (INIFTA-CONICET). The N:P ratio was calculated based on the mass of these dissolved nutrients.

### Nutrient limitation bioassays

Laboratory bioassays were performed with the addition of different sources of P, N and C to evaluate the importance of these nutrients in bacteria and algae growth. Four treatments were performed: Control (no nutrients added), PO_3_ (P addition), N-NH_4_^+^ (ammonium addition), and arginine (organic N and C addition). The ratio of nutrients added in each treatment was done according to Beamud et al. (2010). Incubations were carried out in 500 mL glass bottles, with surface water of the lake (EPI). Three replicates per treatment and control were performed. The flasks were kept at the following controlled conditions: 8°C, 14/10-h light/dark cycle with cool white fluorescent tubes, ~100 μmol photon m^−2^ s^−1^. Four setups were performed to study the nutrient limitation for bacteria, algae, and the competition between them: (a) Bacteria present in the lake water, bacteria counts; (b) Bacteria and Algae combined, bacteria counts; (c) No Bacteria, only Algae, algae counts, and (d) Bacteria and Algae combined, algae count. Bacterial and algal abundances (cells mL^−1^) were determined at the initial day, and at days 3, 5 and 7 of the bioassays. Experimental settings were as follows: (1) Bacteria, lake water was filtered through 3 μm pore membrane filters to eliminate plankton cells >3 μm (procariotyc and eukariotiyc cells); (2) Algae, lake water was filtered through 0.22 μm pore membrane filters to eliminate bacteria cells; (3) Bacteria + Algae, unfiltered lake water and 4 mL of algal inoculum (*ca.* 9,000 cells mL^−1^) of *Keratococcus rhaphidioides*, the only algae species of the phytoplankton present at the moment of the sampling. Bacteria were counted after staining with acridine orange (Hobbie et al., 1977) using an epifluorescence microscope Olympus BX51. Algae were counted under an inverted microscope Leica DM IL LED using Utermöhl technique (Wetzel and Likens, 1991).

### Contribution of sediment bacteria to the release/retention of nutrients bioassays

To evaluate the contribution of sediment bacteria to the release/retention of nutrients, continuous flow experiments were carried out from samples collected [water with Van Dorn bottle and sediments with a surface corer USC 06000 (UWITEC GmbH) in April 2018, in the NA (95 m deep)]. The water and sediment samples were preserved in cold and darkness until the beginning of the experiments, in accordance with the recommendations of APHA (2017). The experiments were carried out, in triplicate, in cylindrical PVC containers with a diameter of 10 cm and a length of 60 cm. In each container, sediment (250 g) and lake water (0.9 L), filtered through 20 and 55 μm pore nets to remove phyto- and zooplankton respectively, were placed in an approximate ratio of 5:3 sediment/water, according to Clavero et al. (1999). Two treatments were established: (1) Control, unsterilized sediment and unfiltered water, and (2) Treatment, unsterilized sediment and filtered water (0.22 μm pore) planktonic prokaryots.

The batches were allowed to stabilize for 10 h to reach the initial equilibrium conditions. All containers were kept at room temperature (20°C), aeriated (using an aerator), and in the dark. The supplied air was maintained sterile using a PES-Syringe prefilter (0.45 μm). The experiments lasted 9 days. At 0 and 9 days of incubation, 500 mL of water were extracted, and the following parameters were measured: T°, pH, conductivity, and redox potential (specific electrodes). Also, the concentrations of dissolved nutrients (PRS and N-NH_4_^+^) were analyzed, according to APHA (2017). Bacterial abundance (cells mL^−1^) was counted as described above. The diversity of microbial communities was determined by sequencing techniques.

### Data analysis

To study the relationships between the different environmental variables studied seasonally at Lake Caviahue (NA and SA) and the bacteria, a Principal Component Analysis (PCA) was carried out. The variables used in the analysis were: temperature, pH, Electrical Conductivity (EC), Dissolved Organic Carbon (DOC), Soluble Reactive Phosphorus (SRP), N-NH_4_^+^, Fe^2+^, Fe^3+^, TFe, SO_4_^2−^, and bacterial abundance, while individuals were the 24 seasonal sampling dates.

To determine treatment effects in the nutrient limitation bioassays performed in the laboratory, a Factorial ANOVA was performed. Two factors were considered: treatment (4 levels: Control, +P, +N, +NC) and time (3 levels: days 0, 3, and 7). The variable under analysis was bacterial biomass. When the results of the ANOVA were significant (5% significance), the appropriate *a posteriori* tests were performed.

The contribution of sediment bacteria to the release/retention of nutrients was evaluated by a Two-way ANOVA with two factors: treatments (2 levels: Control and Treatment) and time (2 levels: days 0 and 9), with 5% significance. The variables under analysis were SRP and N-NH_4_^+^ concentrations and bacterial abundance. When ANOVA results were significant, appropriate *a posteriori* tests were performed.

### DNA isolation, library construction, and sequencing

Filters with bacteria stored at −80°C were used in community DNA extraction. Prior to DNA extraction, the filters were sheared (0.5 mm^2^) with sterile scissors. The pieces obtained from 4 filters were placed in a 2 mL Eppendorf tubes, to which 1 mL of Buffer TE 1X was added. After vigorous vortexing, the cell suspension and the chopped filters were subjected to DNA extraction using Phenol-Chloroform-Isoamyl Alcohol, following the recommendations of Nieto et al. (2009). The DNA obtained was purified using the Genomic DNA Clean and Concentrator^®^ kit (Zymo), quantified by fluorescence using the Quant-iT™ PicoGreen™ dsDNA kit (ThermoFisher), and its quality was verified by spectrometry. High quality DNA was used in the preparation of sequencing libraries, for community analyses.

Amplification of the 16S rDNA V4 region was performed with the primers 515F and 806R in a reaction mixture (final volume 50 μL) consisting of 100 ng of DNA, 10 μM of each primer, 10 mM of dNTPs, and 0.5 μL of Herculase II Fusion DNA Polymerase (Agilent). Amplification conditions used were as follows: initial denaturation at 95°C for 2 min, 30 cycles at 95°C for 20 s, 55°C for 20 s, and 72°C for 30 s, and a final extension at 72°C for 3 min. PCR amplicons were purified with the QIAquick Gel Extraction Kit (Qiagen) and their concentration was determined with the PicoGreen^®^ kit (Turner BioSystems, Inc.). Shotgun and amplicon metagenomic libraries for 150 bp insert size were constructed using the Nextera XT DNA Library Preparation Kit (Illumina). The shotgun DNA library was sequenced on an Illumina HiSeq platform (150 bp paired-end reads) at CD Genomics^1^ in New York, United States. Sequencing throughput achieved was 23 million of PE reads. Amplicon libraries were sequenced at MiSeq platform (150 bp paired-end reads) at INDEAR Argentina. Approximately 0.5 million 150-bp PE reads were generated, with an average of 65 thousand sequence PE reads per sample.

### Sequence manipulations and bioinformatics analyses

The sequence quality was checked using fastqc v0.11.5 (Babraham Bioinformatics, 2019), and adapter removal and trimming was done with fastp v0.23.1 (Chen et al., 2018). The amplicon reads with a > Q20 quality score were retained, then the clean amplicon sequence data were processed using the Amplicon Denoising Algorithm DADA2 (Callahan et al., 2016) using these parameters (−-p-trunc-len-f 300; −-p-trunc-len-r 240; −-p-min-fold-parent-over-abundance 4). The resulting Amplicon Sequence Variants (ASVs) with abundance higher than 1 sequence where taxonomically assigned against the SILVA 16S rRNA database 138 (Yilmaz et al., 2014) using classify-sklearn. Denoising, forward and reverse merging, chimera detection, taxonomic assignment, diversity indexes and rarefaction calculations were done with the platform QIIME2 v2021.8 (Estaki et al., 2020). In the case of the shotgun sequencing, reads with a > Q35 quality score were retained, and *de novo* assembled using SPAdes v3.15.2 (Bankevich et al., 2012) built-in in the pipeline SqueezeMeta v1.5.1 (Tamames and Puente-Sánchez, 2019). Parameters ¨-m sequential -t 90 -a spades -assembly_options ––meta --only-assembler y -t 90¨ were used. Downstream analyses including contigs assembly statistics, ORF prediction and annotation, were performed using the SqueezeMeta built-in software (Laslett and Canback, 2004; Hyatt et al., 2010; Schmieder and Edwards, 2011; Lan et al., 2012; Buchfink et al., 2014; Seeman, 2017) and the following databases: GenBank (Clark et al., 2016), eggNOG (Huerta-Cepas et al., 2017), KEGG (Kanehisa and Subramaniam, 2002), and Pfam (Finn et al., 2014), updated on June of 2022. Plots were done with open-source R v4.2.1, using the following libraries (factoextra, datasets, dplyr, forcats, ggfortify, ggplot2, hrbrthemes, igraph, plotly, tidyr, tidyverse, viridis). Metagenomics sequences used in this study were deposited at the National Center for Biotechnology Information (NCBI) under the BioProject accession ID PRJNA1034071.

## Results

### Physicochemical characterization

**Figure 2:**
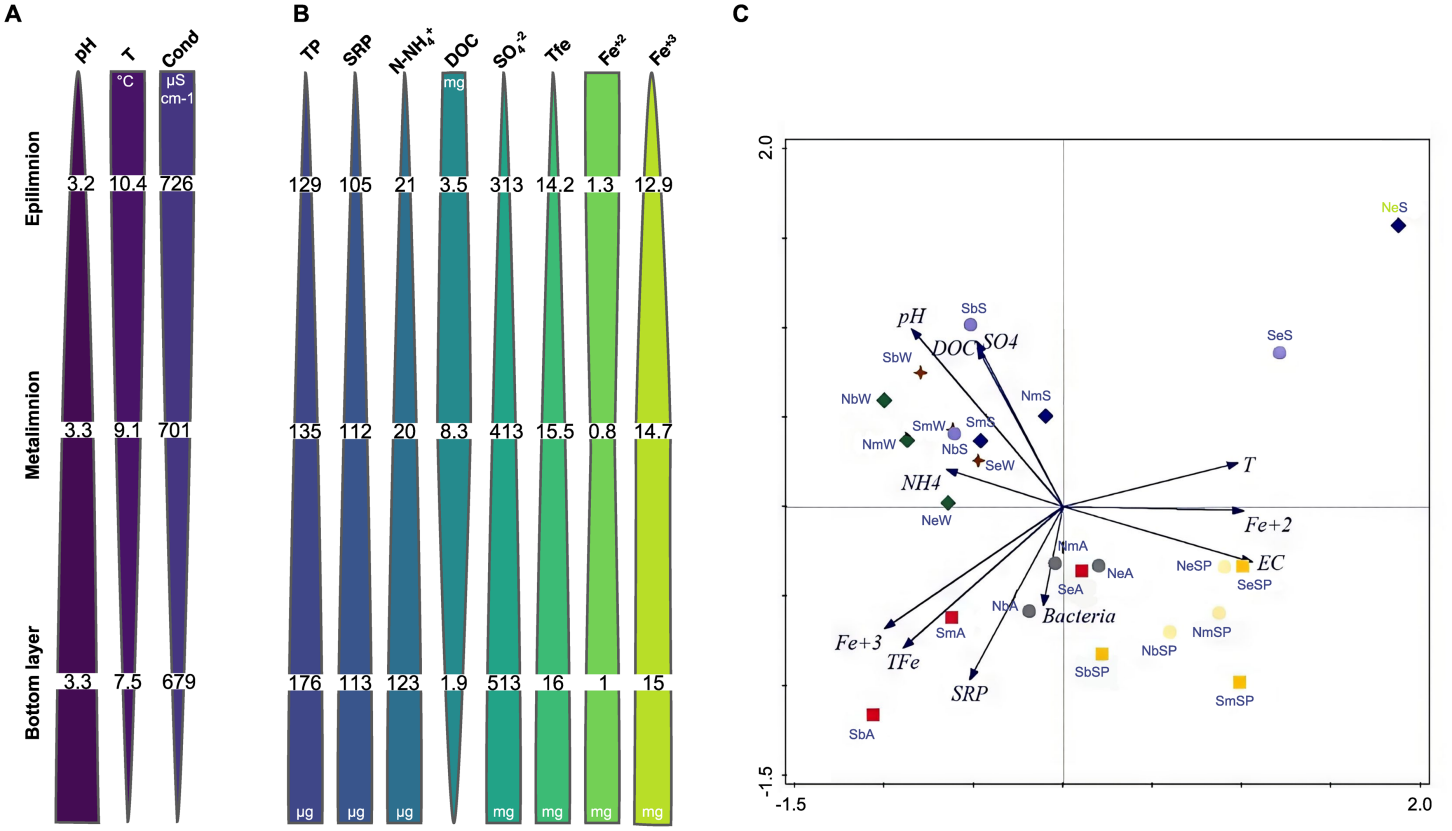
Seasonal average values of physicochemical parameters **(A)** and nutrient concentrations **(B)** in NA of LC in depth: pH, T (temperature, °C), Cond (electrical conductivity, μS.cm^−1^) TP (total phosphorus, μg L^−1^), SRP (soluble reactive phosphorus, μg L^−1^), N-NH_4_^+^ (ammonia, μg L^−1^), DOC (dissolved organic carbon, mg L^−1^), SO_4_^2−^ (sulfates, mg L^−1^), TFe (total iron, mg L^−1^), Fe^2+^ (ferrous ion, mg L^−1^), and Fe^3+^ (ferric ion, mg L^−1^). **(C)** PCA biplot for 24 samples and 11 environmental variables of the Lake Caviahue. Samples and sample abbreviations are described in Supplementary Table 1. Variables: pH, T (temperature), EC (electrical conductivity), SRP (soluble reactive phosphorus), N-NH_4_^+^ (amonia), DOC (dissolved organic carbon), SO_4_^2−^ (sulfates), TFe (total iron), Fe^2+^ (ferrous ion) and Fe^3+^ (ferric ion) and Bacteria. Labels: first letter denote arm of the lake (N, S), second letter is for the depth (e: epilimnion, m: metalimnion and botton) and the third letter is the season (Sp: spring, S: summer, A: autumn and W: winter). NA spring: yellow ■; NA summer: blue ♦; grey ●; green ♦; SA sping: light yellow ●; SA summer: light blue ●; SA autumn: red ■ and SA winter: dark red ♦.

To characterize the current stratification patterns of Lake Caviahue and the changes (physical, chemical and biological) occurring in these patterns according to seasons, the physicochemical characterization of waters was performed. Seasonal average values and ranges of the physico-chemical parameters, nutrient concentrations and bacterial abundance of the water column and sediments samples collected from both arms (NA and SA) of Lake Caviahue (Figure 1), are show in Table 1. Samples designation is summarized in Supplementary Table 1. The average values of the NA parameters for each stratum of the lake are shown in Figure 2.

**Table 1:** Seasonal average values (and ranges) of chemical parameters and bacterial abundance in Lakes Caviahue.

	Strata	pH	Temp (°C)	Cond (μS cm^−1^)	TP (μg L^−1^)	SRP (μg L^−1^)	N-NH_4_^+^ (μg L^−1^)	DOC (mg L^−1^)	SO_4_^2−^ (mg L^−1^)	TFe (mg L^−1^)	Fe^2+^ (mg L^−1^)	Fe^3+^ (mg L^−1^)	Bacterial Abundance
	E	3.2 (3.0–3.3)	10.4 (5.4–14.2)	726 (644–769)	129.2 (120.6–141.5)	105.4 (83.6–127.5)	20.9 (5.8–34.0)	3.4 (1.8–5.0)	312.5 (150–450)	14.2 (7.9–17)	1.3 (0.65–2.02)	12.9 (6.3–16.1)	5.30 (0.48–13.10)
NA	M	3.3 (3.0–3.5)	9.1 (5.6–9.7)	701 (651–736)	134.5 (121.4–151.2)	111.9 (10 1.1–125.3)	19.5 (8.6–27.2)	8.3 (2.2–14.3)	412.5 (100–750)	15.5 (14.4–16.7)	0.8 (0.51–1.37)	14.7 (13.3–16.0)	14.55 (1.24–40.00)
	B	3.3 (3.1–3.5)	7.5 (5.5–9.4)	679 (649–727)	176.2 (117.1–259.5)	113.0 (104.0–123.3)	123.3 (9.4–311.3)	1.9 (0.5–3.3)	512.5 (150–800)	16.0 (15.4–16.9)	1 (0.7–1.76)	15.0 (13.6–16.3)	11.92 (1.51–32.00)
	E	3.2 (3.1–3.3)	10.5 (6.0–14.2)	732 (655–767)	133.3 (122.1–143.6)	110.3 (81.3–125.0)	7.0 (<5–13.2)	10.7 (1.5–19.8)	387.5 (150–600)	15.4 (14–17)	1.3 (0.75–1.8)	14.1 (12.5–16.3)	2.22 (0.20–4.77)
SA	M	3.3 (3.0–3.5)	8.3 (6.0–10.2)	693 (655–740)	158.1 (147.9–171.2)	127.1 (108.7–147.3)	20.7 (<5–55.4)	4.3 (2.0–6.7)	437.5 (100–750)	16.3 (15.2–17.3)	0.9 (0.4–1.8)	15.4 (13.4–16.9)	3.36 (0.69–8.11)
	B	3.4 (3.1–3.6)	7.5 (6.0–8.6)	672 (657–723)	175.1 (133.8–196.0)	117.2 (68.5–165.1)	164.3 (43.7–229.6)	3.4 (1.3–5.4)	556.3 (100–1,400)	16.3 (15.2–18.6)	0.9 (0.5–1.3)	15.4 (13.6–18.1)	24.16 (11.8–37.5)

NA, North Arm; SA, South Arm; E, epilimnion; M, metalimnion; B, Bottom; BA: (× 10^6^ cells mL^−1^).

The pH varied across strata between 3.2 and 3.3 and the water temperature between 7.5 and 10.4°C, showing general stability in both parameters over the sampling periods (Figure 2A). The electrical conductivity (EC) ranged between 644 and 726 μS cm^−1^. The analysis of nutrients revealed a variation in the average total phosphorus (TP) concentration between 129.2 and 173.2 μg L^−1^, of which 74.5% corresponded to the soluble fraction of phosphorus (SRP). The SRP values ranged seasonally between 105.2 and 113.0 μg L^−1^. The average concentration of N-NH_4_^+^ for overall water column in both arms was 62.3 μg L^−1^, varying between <5 μg L^−1^ and 311.3 μg L^−1^. In terms of DOC, the concentration in the NA was, on average, somewhat lower (4.5 μg L^−1^, range: 1.9–8.3 μg L^−1^) than in the SA (6.1 μg L^−1^, range: 3.4–10.7 μg L^−1^) (Figure 2A, Table 1).

Table 1 and Figure 2B show the results of the chemical analyses measured in the water column samples in both arms. The average SO_4_^2−^ concentration was 458 mg L^−1^ for both arms, with minimum and maximum values that varied between 312.5 mg L^−1^ and 512.5 mg L^−1^. Likewise, the average concentration of total iron (TFe) for the water column in both arms was 15.5 mg L^−1^ (14.7 mg L^−1^ for NA and 16.2 mg L^−1^ for SA). The average concentration of Fe^2+^ was very low (1.1 mg L^−1^), representing 8.3% of the TFe. Fe^3+^ represented 93% of TFe, with an average concentration of 14.4 mg L^−1^ (13.6 mg L^−1^ NA and 15.1 mg L^−1^ SA) for the water column in both arms.

Statistical analysis of this data is shown in Figure 2C. The PCA analysis showed that the first two axes explained 60.1% of the total variation of the data. The biplot of samples and environmental variables showed that a first environmental gradient separated the epilimnion samples from the rest by increasing values of temperature, Fe^2+^, EC, and decreasing values of N-NH_4_^+^. Summer samples of the epilimnion were separated from the rest mainly by temperature. The second observed environmental gradient separates the winter and summer samples with high pH and increasing concentrations of N-NH_4_^+^, DOC and SO_4_^2−^ from those of autumn and spring, with high SRP and Fe^3+^ and TFe concentrations. The greatest abundance of bacteria was observed among the autumn samples, regardless of the arm or compartment of the lake sampled, as it was associated with higher values of both dissolved phosphorus and the different forms of iron, and with higher conductivity.

The data obtained indicates that in Lake Caviahue there is a well-established physicochemical vertical gradient of increasing pH, phosphorus, ammonia, total and ferric iron, and sulfate toward the bottom, while temperature, conductivity, dissolved organic carbon and ferrous iron decrease in the same direction (Figure 2). A clear seasonality was observed not only related to temperature, but also to nutrients (N, P and C), bacteria and Fe contents.

### Total biomass and microbial diversity in acidic Lake Caviahue are higher in the bottom strata

**Figure 3:**
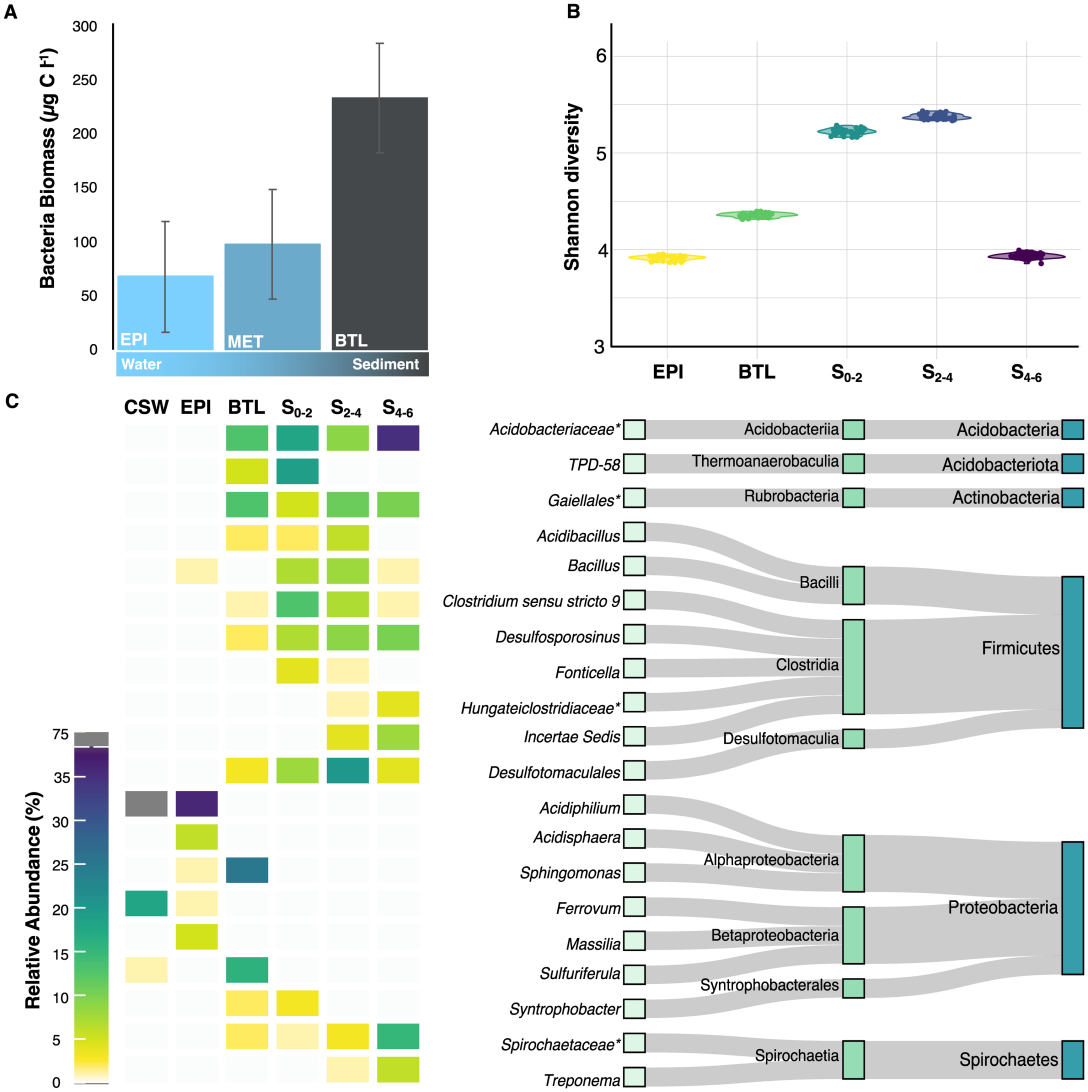
Microbial biomass and community composition at different strata of Lake Caviahue. **(A)** Bacteria biomass (μg C L^−1^) in the water column (epiliminion and metalimnion) and the sediments at the deepest point of the NA of Lake Caviahue. **(B)** Shannon Diversity Index for the microbial communities assayed. **(C)** Relative abundance (%) of the top 20 taxa present in the different strata of Lake Caviahue. Data was derived from targeted metagenomic analyses of the microbial communities in the water column (littoral/costal shallow waters, CSW; the epilimnion at 5 m depth, EPI; the bottom layer water at 95 m depth, BTL) and the sediments (sample at 0–2 cm depth, S0-2; sample at 2–4 cm depth, S2-4; sample at 4–6 cm depth, S4-6). To the right the taxonomy of the top 20 taxa identified is shown (phylum, class, genus).

The average microbial abundance in Lake Caviahue (Table 1) was 12.60 × 10^6^ cells mL^−1^, with higher cell counts in the metalimnion of NA (M, 14.55 10^6^ cells mL^−1^) and the bottom layer of the SA (B, 24.16 × 10^6^ cells mL^−1^). The minimum registered average microbial cells count was in the epilimnion at SA (E, 2.22 × 10^6^ cells mL^−1^) and NA (E, 5.30 × 10^6^ cells mL^−1^). On the other hand, the highest values of bacterial biomass (230 μg C L^−1^) were recovered in the bottom strata samples of both lake arms compared with 75–100 μg C L^−1^ registered in the water column of the lake (Figure 3A) (dF = 2, 21; *F* = 3.9; *p* < 0.05).

To assess how the microbial diversity at each stratum changed with the physicochemical composition of the lake water, we performed targeted metagenomic analyses of littoral samples (CWS), epilimnion, metalimnion and the lake’s sediments (Supplementary Table 2). At sequencing depths greater than 3,000 reads, all rarefaction curves approached asymptotically to the maximum observed number of ASVs for each sample (Supplementary Figure 1). A total of 4,327 different ASVs were obtained, encompassing nearly 162,000 features. All samples used in the study harbored >470 ASVs. A steady increase in the number of ASVs was observed from top to bottom strata. Congruently, the observed richness, the Chao1 estimator, and the diversity indexes (Pielou Evenness, Shannon entropy), were significantly higher in the bottom strata (sediment samples S0-2, S0-4, and S4-6) than in the epilimnion and bottom water column samples (PERMANOVA Bray-Curtis *p* < 0.109, Figure 3B, Supplementary Figure 2). These results are consistent with an increase in the compositional complexity of the microbial communities inhabiting the water samples, along with total biomass, from the surface to the bottom strata of Lake Caviahue.

### Distinct microbial communities inhabit the different strata of Lake Caviahue

An evident change in the dominance profile and complexity of the microbial community was apparent from the comparison of the top 10 most abundant genus-level taxa in the native Lake Caviahue samples (Figure 3C, Supplementary Table 3). The epilimnion community was mainly composed of Fe^3+^ reducing bacteria of the genus *Acidiphilium*, with a relative abundance of 66% on the coastal shallow water column (CSW) and 48% in the NA midpoint at 5 m depth (EPI). A sharp decline in the relative abundance of this taxon was observed toward the bottom layer (BTL) of the NA, completely disappearing from the microbial community in sediments (S). Together with *Acidiphilium*, the Fe^2+^ oxidizer *Ferrovum* occurred at a 30 and 1% relative abundance on the coastal waters and epilimnion, respectively.

In the sediment samples a more complex community was observed, with the presence of up to 10 genera of relative abundance larger than 1%, and many more of lower abundance (28 genus-level ASVs with relative abundance larger than 0.1%). A noticeable increase in the relative abundance of certain genus-level taxa was observed in the sediment fraction, from top (at 0–2 cm, S_0-2_) to bottom (at 4–6 cm, S_4-6_). Nine distinct amplicon sequence variants (ASVs), each with a relative abundance greater than 1% were identified, including representatives of the *Acidobacteriaceae* family (8.7 to 34.6%), the *Galiellales* (5.1 to 9.8%), and *Desulfotomaculales* orders (4.5 to 19.8%) and the genus *Desulfosporosinus* (6.6 to 10.0%). In turn, a few taxa showed the opposite trend, declining in relative abundance in the sediment fraction from top (in S_0-2_) to bottom (in S_4-6_). These taxa included *Clostridium sensu stricto* 9 (12.6% on S_0-2_ to 0.5% at S_4-6_) and TPD-58 (18.7% and disappearing deeper). Several of these taxa are Gram positive, facultative, or obligate anaerobes, and spore formers of frequent occurrence in the sediments of freshwater and acidic pit lakes (Ramamoorthy et al., 2006; Sánchez-Andrea et al., 2015).

The water–sediment interface shared taxa with the sediments (*n* = 9) and with the water column (*n* = 2). Out of the 20 dominant taxa in the littoral (*n* = 3) and midpoint epilimnion (*n* = 6) water column samples, and the sediment layers (*n* = 14), the bottom stratum water column-sediment interface shared 11 taxa (*n* = 9 with the sediments; *n* = 2 with the water column). Four taxa were highly abundant in this stratum (*Sphingomonas*, *Sulfuriferula*, *Acidobacteriaceae*, *Gaiellales*), being *Sphingomonas* and *Sulfuriferula* more abundant here than elsewhere in the lake.

### Epilimnion microbial communities of Lake Caviahue are limited by carbon and nitrogen

**Figure 4:**
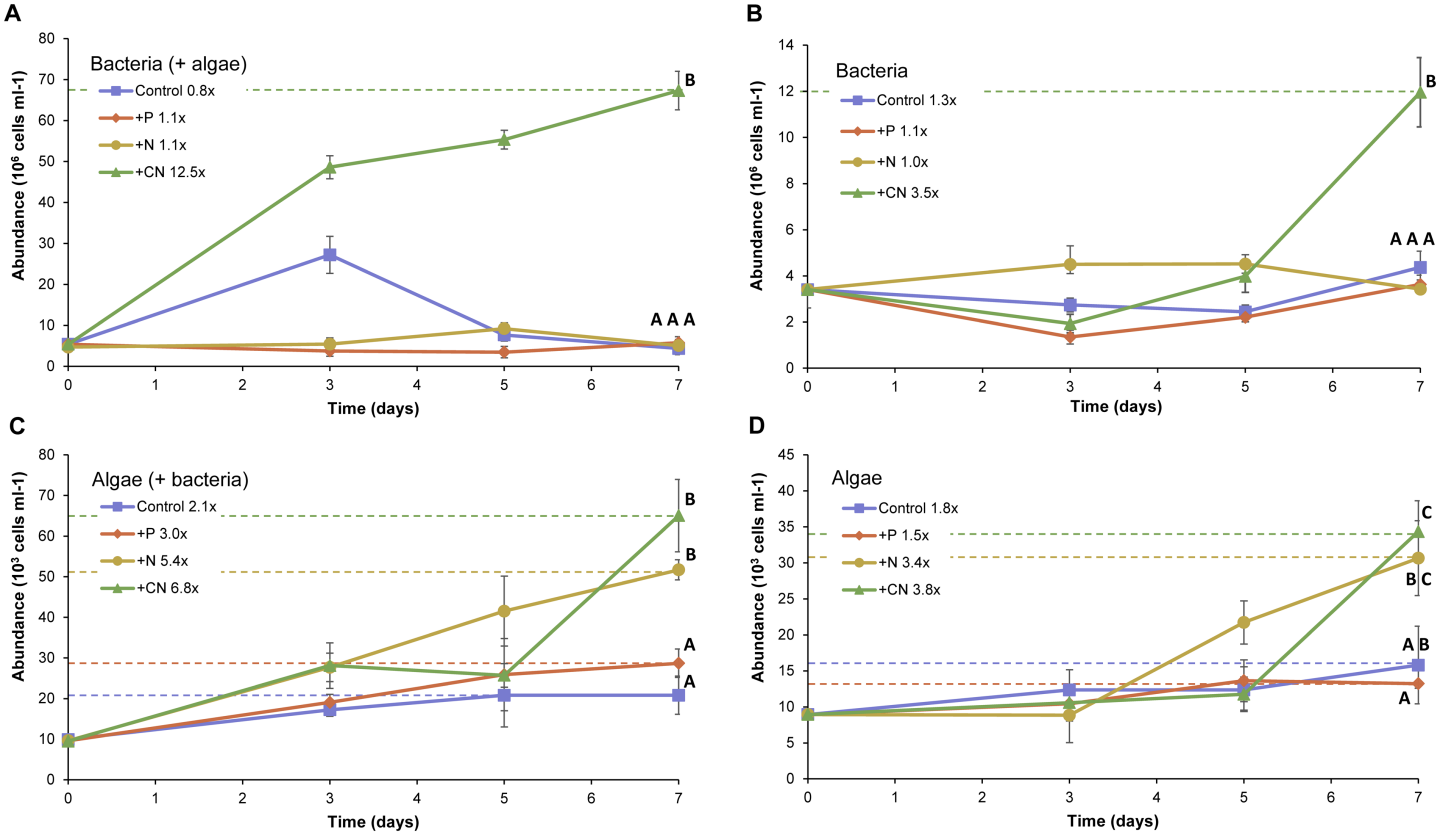
Effect of nutrients amendment in bacterial and algal growth. Bacterial abundance (times 10^6^ cells mL^−1^) in the presence of algae from the Lake Caviahue water sample **(A)**, or in its absence **(B)**. Algae (*Keratococcus raphidioides*) abundance (times 10^3^ cells mL^−1^) in the presence of bacteria from the Lake Caviahue water sample **(C)**, or in its absence **(D)**. The enrichment assays lasted 7 days. All treatments were done in triplicate and are symbolized as follows: control (no nutrients added): ■ blue line; PO_3_ added: ♦ red line; N-NH_4_^+^ added:▼yellow line; Arginine added:▲green line. Fold increase in either bacterial or algal biomass at the end of the experiment is indicated for each treatment (with respect to the control). Cell counts were done as follows: **(A)** Bacteria present in the lake water, bacteria counts; **(B)** Bacteria and Algae combined, bacteria counts; **(C)** Algae present in the lake water, algae counts; **(D)** Algae and Bacteria present in the lake water, algae counts. Cell counts achieved at day 7 for the different tests and treatments were analyzed statistically (ANOVA). Capital letters indicate homogeneous groups resulting from *post hoc* tests.

To assess the role of the water column microbial community (at the epilimnion strata) in nutrient cycling, we evaluated nutrient limitation of growth as proxy. Enrichment assays were performed *in vitro* on freshly sampled native communities and its bacterial and algal fractions recovered by differential filtration. The enrichment assays, amended with sources of phosphate (+P), ammonia (+N), and arginine (+CN), showed that both bacteria and algae (*Keratococcus rhaphidioides*), alone or together, assimilated arginine (Figure 4). In the case of bacteria, the arginine amendment was the single treatment that produced significant differences in net growth (*p* < 0.05), with a 7 fold increase with respect to the control treatment after 7 days of incubation (Figure 4A). The fold increase produced by arginine was lower in the absence of algae in the growth test (Figure 4B). The other three treatments formed a homogeneous group in the post-hoc ANOVA test. Regardless of the presence (Figure 4C) or absence (Figure 4D) of bacteria, most treatments produced observable effects on the algal growth, as reflected by the net increase in cell counts by the end of the experiment (7 days). Effects were stronger in the presence of bacteria. Under the latter conditions, *K. raphidioides* cells (algae) increased their average cell counts up to 2-fold in the presence of phosphate, 2.5-fold in the presence of ammonia and 3.3-fold in the presence of arginine. The treatments with added phosphorus had milder effects than the other treatments evaluated, reaching final cell abundances similar to those of the control treatment alone. In the ANOVA *a posteriori* test, control and added phosphorus constituted a homogeneous group, significantly different from the homogeneous N-NH_4_^+^ and arginine groups.

### Sediment microorganisms contribute to the release/retention of nutrients in Lake Caviahue

**Figure 5:**
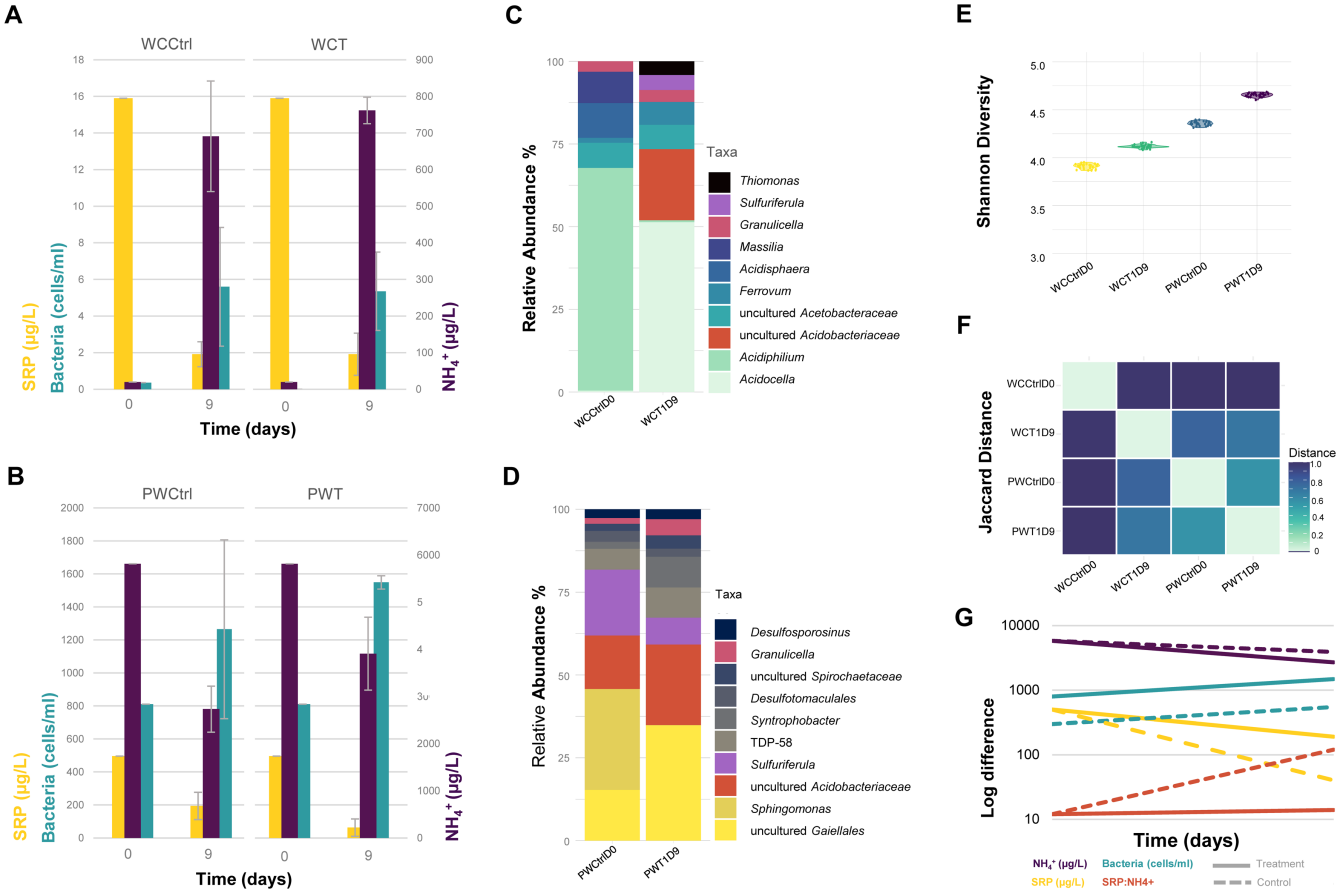
Contribution of sediment and pore water bacterial community to nutrient recirculation of Lake Caviahue. Bacterial abundance (10^6^ cells mL^−1^), SRP (μg L^−1^), and NH_4_^+^ (μg L^−1^) in the control and test columns, for the water column **(A)** and pore water **(B)** fractions, at the start (day 0) and end (day 9) of the bioassay. Relative abundance (%) of the top 10 taxa (genus-level assignment) identified from the 16 rRNA amplicon data for the water column **(C)** and the pore water **(D)** samples, at day 9. **(E)** Shannon Diversity Index for the microbial communities in the control and test columns at day 9, for each assayed fraction (control versus test column). **(F)** Pairwise Jaccard Distance metric calculated for the different samples under comparison to assess their dissimilarity. A distance of 1 indicates high dissimilarity between samples. **(G)** Log fold change in the bacterial abundance, the concentration of N (NH_4_^+^), P (SRP), and the P:N ratio (SRP:NH_4_^+^) at the beginning and the end of the experiment for the control (dashed line) and test (continuous line) treatment.

To evaluate the contribution of sediment and pore water bacteria to the retention and/or release of nutrients in Lake Caviahue, a continuous flow column microcosm bioassay was setup (Supplementary Table 1). The test column consisted of native sediments and epilimnion lake water, filtered to remove microorganisms. The control column consisted of both native sediments and native water. The structure of the microbial community and the concentrations of dissolved nutrient (N-NH_4_^+^ and SRP) in the test column, were evaluated after 9 days and compared to the control. After 10 h of stabilization of the columns, at the onset of the experiment (t_0_), the total microbial cell load in the water was higher in the control column than in the test column (Figure 5A), as expected from the columns initial treatment. Yet, in both test and control microcosms microbial growth was observed (Figures 5A,B), with cell abundances nearly doubling toward the end of the experiment (day 9). Statistical analysis revealed time (days of incubation) as the only significant factor (*p* < 0.05, Table 2). No treatment effect was observed.

**Table 2:** Summary of the ANOVA Table of nutrient limitation bioassays.

	dF	*F*	*p*
Treatment	4	1.1	0.371980
Days	2	6.9	**0.003437**
Treatment*Days	8	2.3	**0.048624**

Values in bold indicate *p* < 0.05.

Targeted metagenomic analysis of the microbial communities in the microcosm’s fractions (water column and pore water) revealed neat differences in structure (Figures 5C,D) and diversity (Figure 5E), both within and between microcosms. A total of 42 ASVs had pooled abundances above 1% in the fractions of interest, consisting mainly of cultured and uncultured taxa of the families *Acetobacteraceae*, *Gaiellaceae*, *Acidobacteriaceae*, *Sulfuricellaceae*, and *Thermoanaerobaculaceae* (Figures 5C,D; Supplementary Table 4). These, and other less abundant taxa, distributed differentially between fractions and microcosms. The Jaccard index for all pairwise AVSs concurrencies shown in Figure 5F, revealed larger dissimilarities between fractions per microcosm (water column versus pore water), than between microcosms (control versus test column). Congruently, we measured higher Shannon’s diversity in the pore water relative to the water column fraction, in both test and control microcosms (Figure 5E). Species diversity in the water fraction of the test column increased with respect to the control, implying that even if water filtration had an impact on the microbial biomass of this fraction at the onset of the experiment, the native sediment’s community contributed to the diversity of the water column after 9 days of incubation.

A small number of taxa occurred exclusively in the water (e.g., *Acidiphilium*) or the pore water fractions (e.g., *Sphingomonas*), at both high and low relative abundances (Supplementary Table 4). While *Acidocella* (*Acetobacteraceae*) was the most abundant genus in the water fraction (epilimnion) of the test microcosm (relative abundance of 42.5% after 9 incubation days), uncultured actinomycetes of the *Gaiellales* order (24.3%; 12.8%) and uncultured bacteria of the *Acidobacteriaceae* family (17.0%; 13.4%) had a neat dominance over the other microorganisms in the pore water from the test and control microcosms, respectively. The community structure of the pore water fractions in both microcosms was generally conserved, with variations in the relative abundance of most taxa not exceeding 1% (data not shown).

Variations between test and control water fractions were more significant, as expected from the initial treatment of the water (filtration). Taxa (ASVs) experiencing the largest variations in abundance between the PW and WC fractions in the test microcosms were uncultured actinomycetes of the *Gaiellales* order (16 fold) and *Thermoanaerobaculaceae* TDP-58 (10 fold), and between the WC and PW fractions *Acidocella* (14 fold) and uncultured *Acetobacteraceae* family (15 fold), originating from the sediments and the epi/hypolimnion, respectively (Supplementary Table 4). In the control microcosms the ASVs that varied most sharply in abundance were *Acidiphilium* (>49 fold between the WC and PW), *Sphigomonas* (27 fold between the PW and WC) and *Sulfuriferula* (>16.5 34 fold between the PW and WC).

Concentrations of dissolved nutrients (SRP and N-NH_4_^+^) in the test and control columns at the onset of the experiment (t0) and after 9 days of incubation (t9) were evaluated (Figures 5A,B). A decrease in the concentration of N-NH_4_^+^ of approximately 1.5 and 2 folds, were observed in the test and control microcosms, respectively (Figure 5G, purple). A decreasing trend was also observed when analyzing the concentrations of SRP (Figure 5G, yellow). For this nutrient, a variation of 61% was recorded for the control, and of 93% for the test experiment, with respect to the initial soluble reactive phosphate concentrations measured. When assessing the N:P ratio (Figure 5G), no significant variation was observed between t_0_ (12 fold) and t_9_ (12 fold) for the control microcosm. Conversely, in the test microcosm the N:P relation increased 9 fold compared to the control microcosm at the end of the assay (t_9_). All together, these results indicate that ammonia and soluble phosphate were utilized/mobilized by the microbial communities from both microcosms, albeit at different rates.

## Discussion

### Physicochemical and environmental gradients in Lake Caviahue

In contrast to other lakes of glacial origin in the Andean-Patagonian region, Lake Caviahue is best defined by its glacial-volcanic origin (Rapacioli, 1985). Main water inputs are glacial meltwaters from Rio Dulce entering the lake at the North arm, and acidified volcanic fluids from Upper Rio Agrio, discharging at the union of the South and North Lake arms (Cabrera et al., 2020). Because of this dual origin and the resulting chemical characteristics, this lake is unique among Andean lakes, which are usually circumneutral, translucent and oligotrophic (Pedrozo et al., 1993; Diaz et al., 2007), and different from acidic lakes linked to mining activity, which are usually turbid, and both sulfate- and iron-rich (Geller et al., 1998; Blodau, 2006).

According to our results, Lake Caviahue exhibits a well-established physicochemical vertical gradient (in pH, electrical conductivity and nutrients) that generally resembles those described in acidic pit lakes (Hedrich and Schippers, 2016), yet differs from these in several aspects. The sulfate ion concentration in both Lake Caviahue arms was high, stable between seasons, and increasing toward the lake’s bottom. Values obtained were in the range of previous studies in Lake Caviahue (Pedrozo et al., 2008a; Varekamp et al., 2009), yet lower than typical values observed in acidic pit lakes, where sulfate is the dominant solute (Soni et al., 2014). In turn, the average concentration of total iron in the water column in both arms was low, and mostly present as ferric iron (93%). Total iron showed little variation between seasons and lake strata (Lake Caviahue’s range 6.3–16.9 mg L^−1^) and differed strongly from the average concentration ranges reported in mining lakes at the Iberian Pyrite Belt (e.g., 19–36,450 mg L^−1^, Sánchez-España et al., 2008) or the mining district in Germany (e.g., 0,3 y 420 mg L^−1^, Geller et al., 2013).

In Lake Caviahue the pH, temperature, and electrical conductivity, showed only minor changes between lake strata (epi, meta, hypo), the largest difference being recorded between the water column and the sediments. While the pH in the water column remained largely constant between lake strata up to the sediment–water interface, it showed slightly higher average values during the sampling period (pH 3.2–3.3, Table 1) than those reported previously (pH 1.9 in November 1998; pH 2.7 in March 2004, etc. - > 3.4), fitting to the pH rising trend observed for the last 2 decades (Cabrera et al., 2020 and references therein). The pH measurements of the sediments have been reported previously, showing an increase with sediment depth from pH of 3.0 (at 0 cm) to pH 4.0 (at 12 cm), with a similar rate of increase in both NA and SA (Cabrera et al., 2016, 2020). Lake Caviahue had high electric conductivity for the whole duration of the study (726–629 μS cm^−1^ and years) and was comparable to reported values in previous studies (560 to 1,600 μS cm^−1^) (Pedrozo et al., 2001, 2008a; Varekamp, 2008; Cabrera et al., 2016). The same trend was observed for temperature, which ranged from 5.4 to 14.2 in summer in the epiliminion and between 5.5 to 9.4 at the lake bottom, approaching previously reported values (12 to 15°C in the epilimnion, and 8°C in the hypolimnion Beamud et al., 2007).

Our results also showed that DOC, dissolved organic carbon, decreased toward the lake bottom after reaching a maximum concentration value measured in the metalimnion, in agreement with previous studies (Beamud et al., 2007; Baffico et al., 2017). This pattern parallels the behaviour of DOC concentrations found in Cueva de la Mora acid pit lake (Falagán et al., 2014), where maximum DOC concentrations occurred just above the chemocline, linked to the abundance of phytoplankton thriving in a relatively shallow photic zone around 10 m of depth (Diez-Ercilla et al., 2014). The lower limit of the photic zone (1% of surface PAR irradiance) in Lake Caviahue has been located at an approximate depth of 16 m (Beamud et al., 2007), and correlates with the maximal chlorophyll-*a* peak (Beamud et al., 2007; Baffico et al., 2017). In other acidic lakes, this interface between the oxygenated mixolimnion and anoxic monimolimnion (i.e., the chemocline), has been shown to be populated by obligate aerobes (e.g., *Leptospirillum ferrooxidans*), facultative anaerobes (e.g., *Acidithiobacillus ferrooxidans*) and obligate anaerobes (e.g., *Desulfomonile* sp.), suggesting the existence of microenvironments of varying oxygen contents within this zone, where both oxidized and reduced iron (ferric and ferrous) and sulfur (sulfate and hydrogen sulfide) dynamically support microbial growth (Wendt-Potthoff et al., 2012).

### Trophic state of Lake Caviahue

According to the trophic categories based on parameters such as TP, SRP, and Chlorophyll ***a*** (Vollenweider, 1968; Wetzel, 2001), Lake Caviahue is classified as mesotrophic if the concentrations of TP and SRP are considered, and ultraoligotrophic if Chlorophyll ***a*** concentration (<0.5 μg L^−1^, Pedrozo et al., 2001) are taken into account. Total concentrations of nutrients (in phosphorous, nitrogen) measured in Lake Caviahue in this study agree with previous classifications and showed, in general, an increase in their concentrations from top to bottom (27% increase in [TP], 83% increasing in [NH_4_^+^]). Relevant variations in the concentration ranges of measured phosphate anions and ammonium cations were observed in Lake Caviahue, both of which were lower compared to reported values in well-studied acidic pit lakes (Santofimia et al., 2013; Falagán et al., 2014; Ayala-Muñoz et al., 2022a), and exhibited a reversed vertical trend compared to that reported for Cueva de la Mora in Spain (Falagán et al., 2014). In Lake Caviahue concentrations of ammonium, considered as a bioavailable inorganic source of nitrogen, varied one order of magnitude between the epilimnion and the water sediment interphase at the lake’s bottom. This ion showed only modest variations throughout the water column in Cueva de la Mora acidic pit lake (Falagán et al., 2014). In turn, concentrations of phosphate, a macro-nutrient that is often poorly bio-available in oxidized acidic pit lakes water due to its poor solubility, varied only moderately between the epilimnion and the lake’s bed (27% difference), with the steepest change (24% increase in concentration) occurring bellow the chemocline (metalimnion stratum). These results indicate that both ammonium and phosphate bioavailability increase from top to bottom of the lake. Ferric iron concentration in Lake Caviahue showed only a 14% increase toward the lake’s bed, implying that factors other than precipitation control this ion deposition dynamics. Interestingly, measurements of the total phosphate and ferric iron entering the Lake Caviahue via Río Agrio (Temporetti et al., 2019) indicate that nearly 87% of TP, 90% of the SRP, and 77% of the ferric iron is precipitated before reaching lake water at the epilimnion. Congruently, in previous works (Pedrozo et al., 2001, 2008a,b; Cabrera et al., 2016) moderate to high concentrations of phosphate and ammonium have been recorded in Lake Caviahue water and sediment samples.

Although the nutrients (P and N) in the water column are the parameters mostly used to establish the trophic state of an aquatic environment (Organization for Economic Co-operation and Development, 1982; Horne and Goldman, 1994; Wetzel, 2001; Schindler et al., 2008), these suffer significant annual fluctuations (Maassen et al., 2005). In this sense, nutrient contributions from sediments become important, representing the internal loading of an environment (Wetzel, 2001; Kaiserli et al., 2002; Golterman, 2004). This internal load can often determine the degree of eutrophication of a waterbody even when the external contributions of nutrients to that environment have been reduced (Carpenter, 2005). In this way, sediments play an important role in P transformation and accumulation processes in aquatic systems (Kowalczewska-Madura et al., 2007). Several studies have linked the TP concentration in surface sediments to the TP concentration in the water column assuming, theoretically, that trophic state is strongly influenced by sediment P release (Carey and Rydin, 2011). Temporetti et al. (2014) evaluated the distribution of sediment TP concentration and the trophic state of aquatic environments of the Argentinean Patagonia and showed that certain parameters of pore water (SRP concentration) and sediments (P-Labile fraction, depth distribution pattern of TP and Metals:P ratio) were significantly correlated to the trophic state of the environments studied. In this sense, Lake Caviahue is classified as oligotrophic.

### Microbial abundance and diversity and community composition

Results obtained in this study revealed that both average and maximal bacterial abundances (cell counts) per strata were higher in the NA than in the SA, with minimal counts in the epilimnion (2.7 fold lower than in the metalimnion and 2.2 fold lower that in the bottom strata) and maximal total bacterial biomass recovered from the bottom strata (2–3 fold higher than in the water column). These values are consistent with previous ones obtained in Lake Caviahue (Pedrozo et al., 2001) and higher than bacterial biomass values in the shallow (4–14 m depth) acidic mining lakes studied by Kamjunke et al. (2005). In these environments with a pH range between 2.3–3.0, biomass values varied between 4 and 82 μg C L^−1^, while in natural acidic Lake Caviahue the values varied between 75 and 230 μg C L^−1^. Lack of total cell counts or C μg L^−1^ information in reports studying deeper acidic lakes [e.g., in Falagán et al. (2014), Cueva de la Mora lake, 30 m deep and Guadiana lake, 55 mt deep; in Santofimia et al. (2013): Nuestra Señora del Carmen lake, 35 m deep] prevent further direct comparisons of the bacterial biomass variations between natural and mining acidic lakes which are both deep acidic lakes.

Despite the differences in magnitude, or the change of magnitude between strata, Lake Caviahue resembles these other systems in that in all, a considerable increase in microbial diversity from the surface to the bottom layers of the lake is observed. The number of ASVs from Lake Caviahue epi-to-bottom (2.3 fold), were consistent with the observed increase in total biomass. Reconstructed SSU sequences from metagenomic data from a study of Cueva de la Mora, reported a similar increasing trend from top to bottom (2.1 fold) for taxa of relative abundance >1% between the upper oxic layer (3 m) and the deep anoxic layer (35 m) (Ayala-Muñoz et al., 2020). These changes in diversity are likely to parallel changes in abundance/biomass. Also, as in the case of acidic pit lakes, microbial communities shaping the geochemistry of these lakes remain poorly explored (Ayala-Muñoz et al., 2022a).

### Distinct microbial communities inhabit the different strata of Lake Caviahue

**Figure 6:**
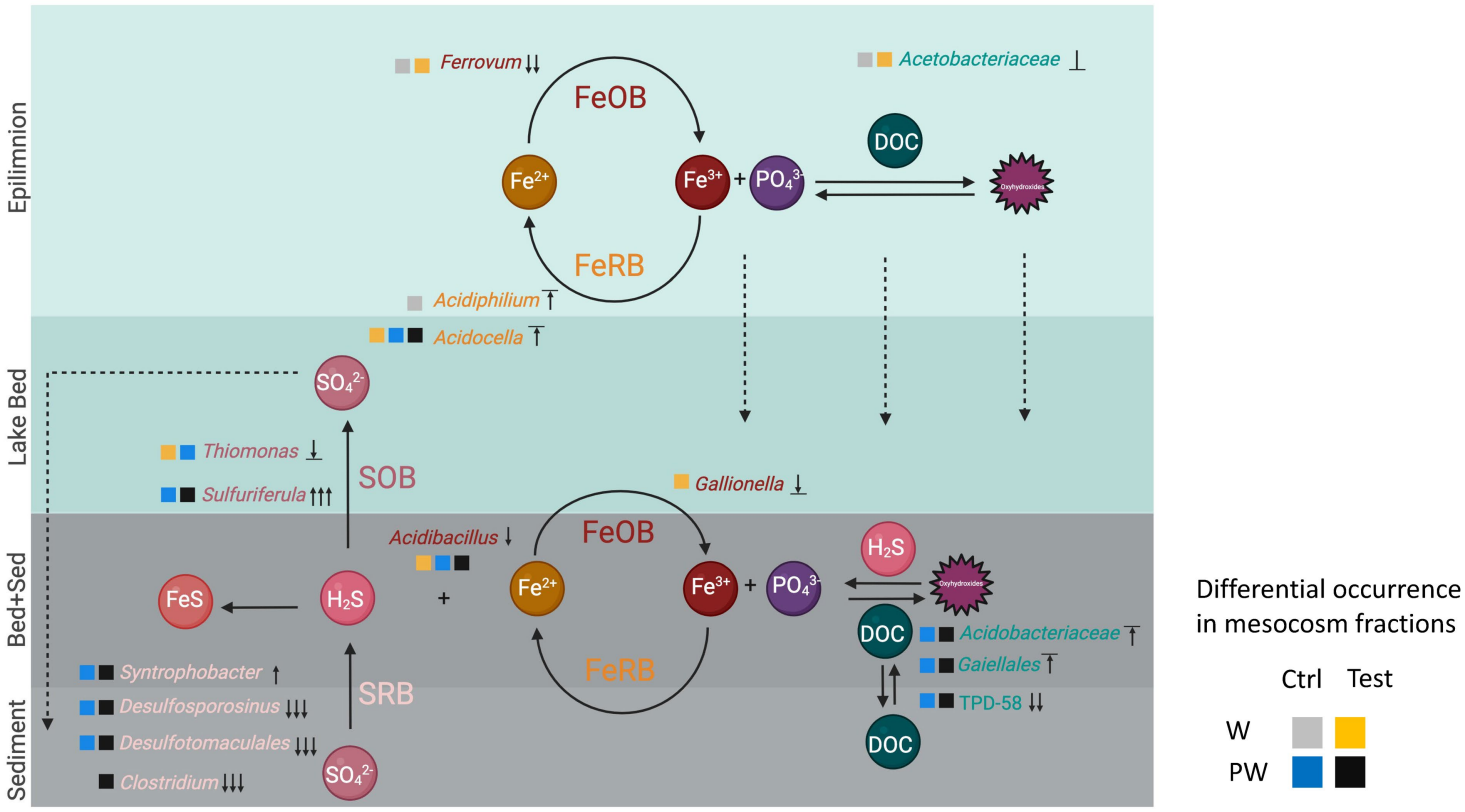
Conceptual model of the biogeochemical cycling of nutrients, sulfur, and iron at the sediment-water interphase of Lake Caviahue. Lake strata are depicted as colored layers and labeled accordingly to the left. Oxic strata are colored in light blue to blue and anoxic strata in grey to dark grey. Nutrients and electron donors and acceptors are shown in circles and a/biotic transformations are depicted by arrows (oxidation, reduction, precipitation). Transformations are inferred on the basis of the occurrence of taxa with known metabolic capacities or pathways according to the literature listed in the discussion. Microbial taxa occurring in each stratum of LC at relative abundance >1% is indicated by the lowest taxonomic rank-available. Occurrence of the taxa in the flow column experiments is indicated by squares, color coded according to the treatment (control or test) and the sample fraction (WC or PW) as follows: WCCtrlD0, grey; WCTD9, yellow; PWCtrlD0, blue; PWTD9, black. Direction of the growth response overtime (day 0 to day 9), assessed on the basis of the change in relative abundance, is symbolized according to its magnitude as follows: increase, ↑ low to medium, ↑↑ medium to high, ↑↑↑ low to high; decrease, ↓ low to medium, ↓↓ medium to high, ↓↓↓ low to high; no change, ‾↑‾ high to high, ⊥ medium to medium, _↓_ low to low. Labels: sulfur oxidizing bacteria (SOB), iron oxidizing bacteria (FeOB), sulfate-reducing bacteria (SRB), iron reducing bacteria (FeRB). Carbon is fixed by algae (Trebouxiophyceae). Disolved organic carbon is degraded by phylogenetically diverse heterotophic and fermentative bacteria including members of the Acetobacteriaceae, Acidobacteriaceae, TPD-58, and Clostridium. Fermentative end-products are linked to respiratory iron reduction by Gaillales, sulfate reduction by Syntrophobacter, Desulfotomaculales, and Desulfosporinus. Sulfide produced can be oxidized by Acidibacillus (Ferracidibacillus gen nov.), Gallionella, or Ferrovum and reduced back to ferrous iron by Acidiphilium and/or Acidocella. This figure was created with BioRender.com.

Our study revealed significant changes in the composition and complexity of the microbial communities across the different strata of Lake Caviahue (Figure 6). In contrast to studies of acidic mine pit lakes, such as Cueva de la Mora, where eukaryotic microorganisms are dominant in the upper layer, bacteria in the chemocline, and archaea in the deep layer (Ayala-Muñoz et al., 2020), in LC no archaeal groups were detected at relative abundances above 1% in any of the strata analyzed, regardless of their occurrence in the stream waters entering the lake (Lopez Bedogni et al., 2020). Even if this study did not target eukaryotes, previous work at Lake Caviahue has shown that this domain is present in the largest abundances in the surface layer of LC (>90% abundance), consisting mostly of *Keratococcus raphidioides* algae (Beamud et al., 2007, 2010), along with bdelloid rotifers (Pedrozo et al., 2001), while some diatoms have been recovered from the lake sediments (Baffico et al., 2017).

The primary member of the microbial community of surface waters at the Lake Caviahue NA were bacteria of the genus *Acidiphilium* with a relative abundance of 66% on the coastal water column at 0.5 m depth, and 49% in the NA midpoint at 5 m depth. This Fe^3+^ reducing chemo-organotroph (Küsel et al., 1999; Li et al., 2020) is a frequent member of the microbial communities in acidic mineral environments, such as acid mine drainages (Harrison, 1981; Wakao et al., 1994), and is also prevalent in acidic pit lakes (Falagán et al., 2014; Ayala-Muñoz et al., 2020) and natural acidic lakes, such as the crater lake of the active Poás Volcano in Costa Rica (Wang et al., 2022). Being heterotrophs and facultative anaerobes, they are key players in the iron cycle in this stratum of Lake Caviahue. Despite their abundance, evidence generated in the nutrient limitation bioassays performed indicates that the bacterial populations are limited by C and N. The arginine amendment treatment produced a net increase in the biomass of epilimnion bacteria with respect to the control after a week’s time, indicating the capacity the taxa present to use arginine for growth, either as sole C source or in the presence of algal exudates. Based on *Acidiphilium* spp. dominance in this stratum, it is likely that the observed growth corresponded to that of this taxon. Literature evidence supports the capacity of several *Acidiphilium* spp. to utilize arginine as C source under heterotrophic growth conditions, including *Acidiphilium cryptum*, *Acidiphilium multivorum*, and *Acidiphilium organivorum* (Hiraishi and Imhoff, 2015). The 16S rRNA amplicons recovered from Lake Caviahue could be affiliated to Group Ia isolates (Okamura et al., 2015), having a 100% sequence identity with *Acidiphilium rubrum/angustum* and 99% identity with *Acidiphilium iwatense.* Arginine consumption for most Group Ia isolates remains to be tested, excepting *Acidiphilium angustum/rubrum* strain ATCC 35905 which was reported unable to use arginine as C-N source (Hiraishi and Imhoff, 2015). Yet, our results strongly suggest Lake Caviahue Group Ia *Acidiphilium* spp. could have this capacity. Alternatively, the biomass increase could be due to concurrent growth of *Acidocella* spp., present in the water sample at lower relative abundances (and seen to take over in continuous flow column experiments). Species of this *Acetobacteraceae* family genus (e.g., *Acidocella aminolytica*) can also use L-arginine as a sole carbon source (Kishimoto et al., 1993, 1995). Furthermore, both *Acidiphilium* and *Acidocella* species are recognized to concur and interact with Chlorophyta division algae, such as *Chlamydomonadaceae* (Ňancucheo and Johnson, 2012 and *Trebouxiophyceae* Servín-Garcidueñas and Martínez-Romero, 2012), either *in situ* or *in vitro.* Algae exudates have been shown to have a role in sustaining populations of acidophilic heterotrophic bacteria by providing them with organic substrates. These is in line with the stronger effects on bacterial growth observed in our amendment tests by the presence of the algal fraction. Monosaccharides and sugar alcohols, some of which are produced and exudated by algae (Ňancucheo and Johnson, 2012), have indeed been shown to be suitable carbon and energy sources for these species (Okamura et al., 2015). In turn, the end product of bacterial C metabolism, CO_2_, might benefit the algae, shaping a syntrophic interaction (Johnson, 2016). This would explain the net increase in growth of the bacterial biomass in the presence of the algae, and the reciprocate effect observed by the presence of bacteria on algae.

Together with *Acidiphilium*, *Ferrovum* spp. where the only other bacterial taxa occurring at significant abundances in the surperficial lake waters, particularly at coastal waters (30%). A sharp decline in the relative abundance of this taxon was observed toward the epiliminion (1%) and the bottom layer of the NA of Lake Caviahue (5.8%), completely disappearing from the microbial community in the sediments. This behaviour cannot be explained by the observed changes in the ferric-ferrous iron concentrations, which remained low and constant from the upper layer, through the thermocline, and toward the bottom layer. In turn, it correlates with the dissolved oxygen availability (reported in Beamud et al., 2007) and follows the trend of chlorophyl *a*, which declines bellow 20 m of depth. Concurrence of *Acidiphilium* and *Ferrovum* has been reported in acidic pit lakes at the Nanshan iron mine in China (Hao et al., 2017; She et al., 2023) and streams draining the abandoned Mynydd Parys copper mine in north Wales (Johnson et al., 2014), under conditions in which one drives the oxidation of ferrous iron and the other drives the reduction of ferric iron. *Ferrovum myxofaciens* is described as an extremely acidophilic, psychrotolerant, autotrophic, and obligate ferrous iron oxidizer, which requires oxygen as electron acceptor for growth (Johnson et al., 2014). In turn, group Ia *Acidiphilium* spp. are capable of semi-anaerobic growth coupled with reduction of Fe^3+^ (Okamura et al., 2015), as other species of the genus and family excepting *Acidisphaera*-like isolates (Coupland and Johnson, 2008). This, suggests that *Acidiphilium* abundance patterns correlate with the availability of algae derived nutrients, and electron acceptors (Fe^3+^) generated abiotically by the volcanic iron inputs entering the lake via the Rio Agrio stream waters, and biotically by iron oxidizers present in the lake, mostly *Ferrovum* spp. The latter, in turn, partition following oxygen availability. The slight increase in abundance of *Ferrovum* spp. toward the lake bed can possibly be explained by precipitation processes of cell aggregates and filaments, referred to as streamers, known to be copiously produced by this taxon (Kimura et al., 2011; Johnson, 2016). Altogether, the evidence collected, suggests that electron acceptors are the main drivers of microbial niche partitioning in the water column strata of Lake Caviahue. This has been suggested previously in other aquatic environments (e.g., Martín-Rodríguez, 2023). The close interaction between *Acidiphilum* and *Ferrovum* species may also result from increased local availability of CO_2_ as a by-product of heterotrophic metabolism by *Acidiphilium* and its growth promoting effect on iron-oxidizing bacteria such as *Ferrovum* (Kermer et al., 2012; Mosler et al., 2013).

Community profiling analyses performed, also showed the increase in abundance toward the lake bed (B, b, Bottom) of *Acidocella* (up to 42% of the community >1%), uncultured bacteria of *Acidobacteriaceae* (up to 17% of the community >1%) and *Acetobacteraceae* (up to 5.9% of the community >1%). These taxa are all versatile heterotrophs that can be linked to the breakdown of organic compounds and carbon cycling in deep layers of acidic pit lakes such as Cueva de la Mora (Ayala-Muñoz et al., 2022a) and Guadiana pit lake (Falagán et al., 2014), which is likely also their role in the hypolimnion of Lake Caviahue. Sulfur and iron cycling microorganisms such as *Sulfuriferula*, *Thiomonas* and *Acidibacillus* (approximately 3% of the community each) were also fairly abundant toward the lake bottom. *Sulfuriferula*-like bacteria (Betaproteobacteria) have been identified as part of the microbial community at 30 m depth in the Iberian Pyrite Belt acidic pit lake Filón Centro (van der van der Graaf et al., 2020). They can grow autotrophically on inorganic sulfur compounds, and heterotrophically on a number of organic substrates (including complex organic substrates, sugars, organic acids and an alcohol), in the absence of sulfur compounds as an electron donor (Drobner et al., 1992; Watanabe et al., 2015). Members of the genus *Thiomonas* (Betaproteobacteria) are mixotrophic sulfur oxidizers (Chen et al., 2004), yet some species can also oxidize Fe^2+^ (Coupland et al., 2004; Battaglia-Brunet et al., 2006). They are frequent inhabitants in slightly acidic and sulfidic sediments that result as AMD waters discharge across diverse landscapes (Akob et al., 2020), and have also been found at oxygen-depleted layers of acidic pit lakes (e.g., at Brunita, Spain; Sánchez-España et al., 2020). Although their known ecological roles are mostly linked to aerobic oxidation of sulfur, iron and arsenite (in the presence of low concentrations of organic carbon) genomic studies have suggested some species may use nitrate anaerobically (Arsène-Ploetze et al., 2010). Known species of *Acidibacillus* (Firmicutes) are facultative chemolitho-heterotrophs, that require an organic carbon source and oxidize both ferrous iron and elemental sulfur at moderately high temperatures (43°C), or only ferrous iron at mesophilic temperatures (30°C) (Holanda et al., 2016). All isolates characterized to date have been shown capable of dissimilatory ferric iron reduction under anaerobic conditions. Members of this genus have been shown to be present in low abundance (2.8%) at the acidic pit lake at Sherlovaya Gora (pH < 4.9) (Gavrilov et al., 2019). All three taxa (*Sulfuriferula*, *Thiomonas*, and *Acidibacillus*) have in common their ability to switch between different metabolic pathways depending on the availability of specific substrates and environmental conditions, linking sulfur and iron cycles (used as energy sources) with organic compounds cycling (used either as electron donors, carbon sources, or both). They can thus influence the decomposition and mineralization of organic matter in the deeper strata of Lake Caviahue.

At the interface between the lake bed and the sediments a different group of bacteria seem to take over this role. Amplicon sequence data obtained in this study, indicates that highly abundant uncultivated microorganisms of the *Thermoanaerobaculaceae* (TDP-58), *Acidobacteriaceae* (subgroup_1), and *Gaiellaceae* families populate Lake Caviahue’s sediments, and the pore water in the flow column bioassays. Related microorganisms have been reported in anoxic or low-oxygenated bottom layers of lakes both freshwater and acidic lakes. The family *Thermoanaerobaculaceae* (Dedysh and Yilmaz, 2018), has only one validly described representative, *Thermoanaerobaculum aquaticum* MP-01 T. This strain was isolated from a freshwater hot spring and is a strictly anaerobic and neutrophilic thermophile capable of fermentative growth, and both Fe^3+^ and Mn^4+^ reduction (Losey et al., 2013). Even if TPD-58 is proposed as a different genus of this family, best guess is it could have similar metabolic roles in Lake Caviahue sediments. *Acidobacteriaceae* uncultured representatives found in the lake bed and sediments pertained to subdivision 1 (represented by *A. capsulatum*, Hiraishi et al., 1995), a poorly defined taxon encompassing at least 11 genera (Kielak et al., 2016). Its members are physiologically diverse and have been found to dominate in low pH conditions (Sait et al., 2006). They are described as oligotrophic and psychrotolerant bacteria, with some preference for CO_2_ (Stevenson et al., 2004) or low pH (Sait et al., 2006). They have been found to thrive in acid mine drainage waters (Hallberg and Johnson, 2003; Hallberg et al., 2006) and in acidic pit lakes (Kleinsteuber et al., 2008). Evidence obtained in lake 111, a shallow yet stratified mining lake, supports the distribution of *Acidobacteriaceae* in the hypolimnion, where CO_2_ concentrations measured as total inorganic carbon are higher (around 10 mg L^−1^) than in the epilimnion (below 1 mg L^−1^), and pH is mildly acidic (Herzsprung et al., 1998; Zippel et al., 2001). Also, isolates recovered from acid mine drainage waters have been shown to grow better under microaerobic conditions, suggesting a role in the acceleration of the reductive dissolution of ferric minerals (Hallberg and Johnson, 2003; Malik and Hedrich, 2022). These conjunct characteristics, acid tolerance, microaerobicity and CO_2_ requirement, could explain the occurrence and abundance profile of this taxon in the LC lake bed. Members of the family *Gaiellaceae* (*Actinobacteria*) are strictly aerobic and chemoorganotrophic mesophiles (Albuquerque et al., 2011), so far represented by a single cultured specie, *Gaiella occulta*. The type strain (F2-233^T^) was isolated from a 150 meter deep water aquifer in Portugal (Albuquerque et al., 2011), and other representatives have been traced to soils (GenBank LSTI01000000), karst systems (Zhu et al., 2019) and volcanic caves (Riquelme et al., 2015), supporting their general existence in subterranean environments. Little else is known about their ecophysiology, apart from the fact that they seem to be key players of the nitrogen cycle in these environments, participating in the conversion of nitrate to nitrite (Albuquerque et al., 2011). For this role, they are thus considered keystone members in the bacterial networks of caves (Zhu et al., 2019; Ma et al., 2021).

The microbial community of the sediments from Lake Caviahue was complex and differences in the taxa profiles were observed between depths. In addition to the *Acidobacteriaceae* family (8.7 to 34.6%) and the *Galiellaceae* (5.1 to 9.8%) found at the interface between the lake bed and the sediments, another 12 bacterial families with relative abundance larger than 1% occurred in the sediment cores evaluated. Of these, 10 pertained to the Firmicutes phylum, and occurred in the sediment fractions at high (e.g., families of *Desulfotomaculales*), medium (e.g., *Ethanoligenenaceae*) and low (e.g., *Planococcaceae*) pooled abundance, with 20% of the total ASVs pertaining to taxa of abundance lower that 1%. The Firmicutes are an extremely diverse phylum of bacteria, with a plethora of possible ecological roles. Acid-tolerant, sulfate-reducing and sulfide-producing Firmicutes have been described previously (Holanda et al., 2016; Holanda and Johnson, 2020; Johnson et al., 2023), yet many remain uncharacterized. In common, most Firmicutes are endospores formers (Galperin, 2013), so it is likely that several of the taxa identified in the sediments of Lake Caviahue are dormant and/or metabolically inactive cells. Yet, 4 of the families present in the native sediment of Lake Caviahue were also identified in the pore water in the continuous flow column bioassays, supporting their active participation in sediment’s nutrient cycling. Their generalized capacity of fermentation or anaerobic respiration would support their participation in the decomposition of organic matter accumulated in the sediment fractions (Zhao et al., 2017).

Differences in the taxa profiles were observed between fractions, the largest being attributable to highly abundant taxa. The dominant taxon in the upper fraction of the sediments was TPD-85 (*Thermoabaerbaculaceae*; 18.7%), followed closely by *Clostridiaceae* (Clostridium_sensu_stricto_9; 12.5% total relative abundance). In turn, the top ranking taxa at the deeper sediment fractions were the *Desulfotomaculales* (at the 2 to 4 cm fraction S_2-4_) and the *Desulfitobacteriales*, represented by members of the *Desulfosporosinus* genus (at the 4 to 6 cm fraction S_4-6_). Both taxa are strict anaerobes and play a roles in sulfur cycling, contributing to the reduction of sulfate (Stackebrandt et al., 1997; Spring and Rosenzweig, 2006; Watanabe et al., 2020). The *Desulfotomaculales* are chemoorganoheterotrophs which grow using organic acids, alcohols, amino acids, and sugars, and also chemolithoheterotrophs which grow using H_2_ and CO_2_ in the presence of acetate, using sulfate, sulfite, and thiosulfate as electron acceptors, which are then reduced to H_2_S (Watanabe et al., 2020). *Desulfosporosinus* spp. are capable of the fermentation of pyruvate, incomplete oxidation of substrates to acetate, and utilization of thiosulfate as terminal electron acceptor. They also have the ability to grow autotrophically with H_2_, via fermentation of lactate, and utilization of sulfate as terminal electron acceptor (Spring and Rosenzweig, 2006). For their characteristics, these anaerobes are restricted to anoxic water-saturated soil environments and freshwater sediments, being frequent in sediments from mine sites and acid mine drainages (Alazard et al., 2010; Sánchez-Andrea et al., 2015; Mardanov et al., 2016; Panova et al., 2021). Their environmental significance in these habitats, and likely also in Lake Caviahue sediments, is the production of sulfide (biosulfidogenesis, Ayala-Muñoz et al., 2022b), which can contribute to acid neutralization through the consumption of protons (Johnson and Sánchez-Andrea, 2019) and the sequestration of metals into highly insoluble metal sulfides (Ňancucheo and Johnson, 2012), both of which are highly beneficial for the remediation of acidic mine/rock drainage systems (Johnson and Santos, 2020).

Uncultivability and limited understanding of the metabolism of many of the bacteria identified in the sediments prevent us from establishing a direct link between their occurrence and abundance patterns with the phosphorous biogeochemical cycling in Lake Caviahue. Yet, taxa profile changes observed in the flow column microcosms over time, which accompanied a neat decrease in the concentration of dissolved nutrients SRP and N-NH_4_^+^, indicates that the microbial communities in the sediments contribute to the retention and/or release of nutrients in Lake Caviahue, and strongly points to uncultured *Gaiellales* (11.6% increase), *Syntrophobacter* (4.7% increase) and subgroup 1 *Acidobacteriaceae* (3.6% increase) as key players in ammonia and soluble phosphate mobilization at the sediment water interface. All together, these results indicate that sediment and microbial communities are active contributors to the nutrient structure dynamics at the water–sediment interface in this extremely acidic glacial-volcanic lake.

## Conclusion

The pH and temperature of Lake Caviahue are similar between arms and are essentially stable between lake strata, with seasonal variations that give rise to two major environmental gradients: one distinguishing the surface water samples due to temperature, Fe^2+^, and electrical conductivity, and the other distinguishing winter and summer samples due to the high pH and increasing concentrations of N-NH_4_^+^, DOC, and SO_4_^2−^ from autumn and spring samples with high soluble reactive phosphorus (SRP) and iron concentrations. The largest bacterial abundance was found in autumn, alongside higher levels of dissolved phosphorus, iron forms, and increased conductivity. Regardless of the season, the highest values of bacterial biomass and ASVs counts were found in the bottom strata of the lake, which is also where the greatest diversity in microbial communities was found. In the epilimnion strata, nutrient cycling was found to be influenced by both bacteria (*Acidiphilium* and *Ferrovum*) and algae (*Keratococcus rhaphidioides*), both of which are limited by CN according to enrichment assays. Arginine amendment significantly increased bacterial growth and impacted the growth of algae, especially when both concurred, which is congruent with a potential syntrophic relationship between the bacteria and algae. The experiments using continuous flow column microcosms showed that microbial growth over time in both the test and control columns, was accompanied by a decrease in the concentration of dissolved nutrients (SRP and N-NH_4_^+^), providing proof that sediment microorganisms are active and contribute significantly to nutrient utilization/mobilization.

## Data availability statement

The data presented in the study are deposited in the BioProject database, accession number PRJNA1034071 and the Sequence Read Archive (SRA) repository, accession numbers SRR26596989 to SRR26596996.

## Author contributions

MC: Formal analysis, Investigation, Methodology, Writing – original draft. IF: Formal analysis, Investigation, Methodology, Writing – review & editing. FD-G: Formal analysis, Writing – review & editing. MD: Conceptualization, Formal analysis, Investigation, Supervision, Writing – original draft, Writing – review & editing. RQ: Conceptualization, Formal analysis, Funding acquisition, Investigation, Methodology, Project administration, Resources, Supervision, Writing – original draft, Writing – review & editing. GB: Formal analysis, Investigation, Methodology, Software, Writing – review & editing. FP: Writing – review & editing. PT: Conceptualization, Formal analysis, Funding acquisition, Investigation, Methodology, Project administration, Resources, Supervision, Writing – original draft, Writing – review & editing.

## Footnotes

^1^ https://www.cd-genomics.com
