# Supplementary figures and images for: Nutrient structure dynamics and microbial communities at the water–sediment interface in an extremely acidic lake in northern Patagonia
Source: Front Microbiol. 2024 Feb 12;15:1335978. doi: 10.3389/fmicb.2024.1335978 (PMC10895001; doi:10.3389/fmicb.2024.1335978)

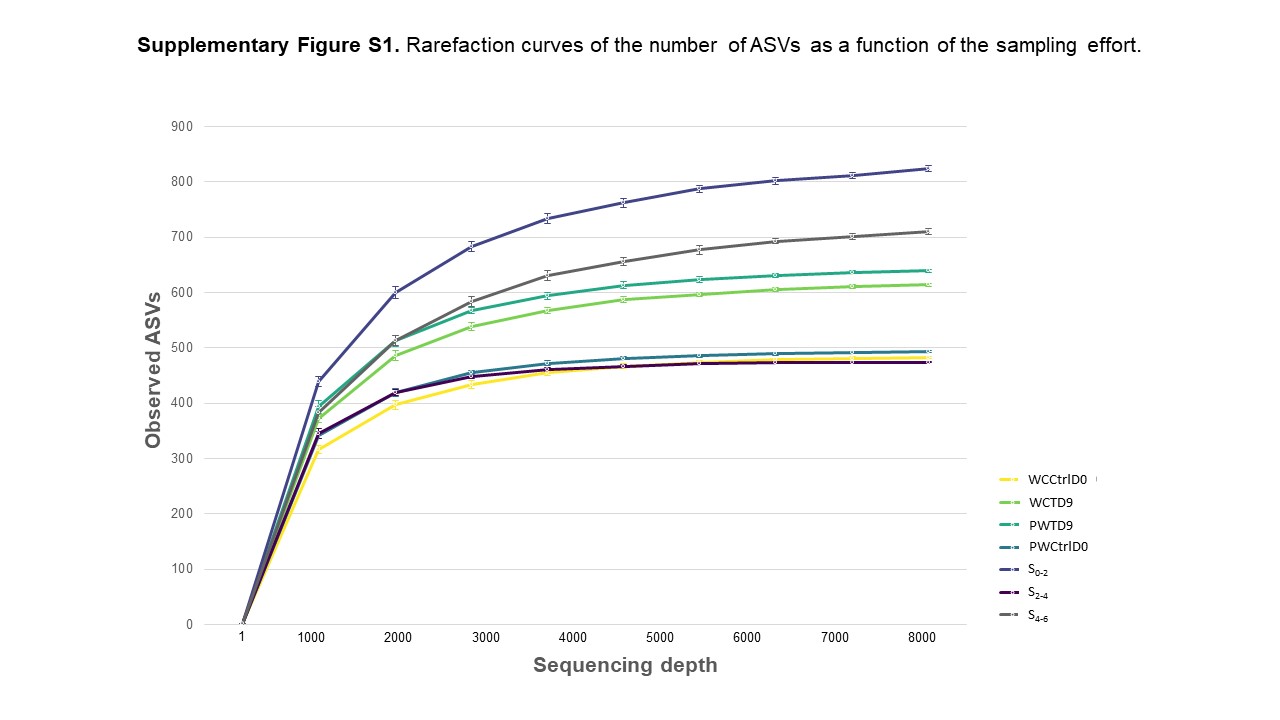

Supplement: Supplementary file 5 [file Image_1.JPEG]

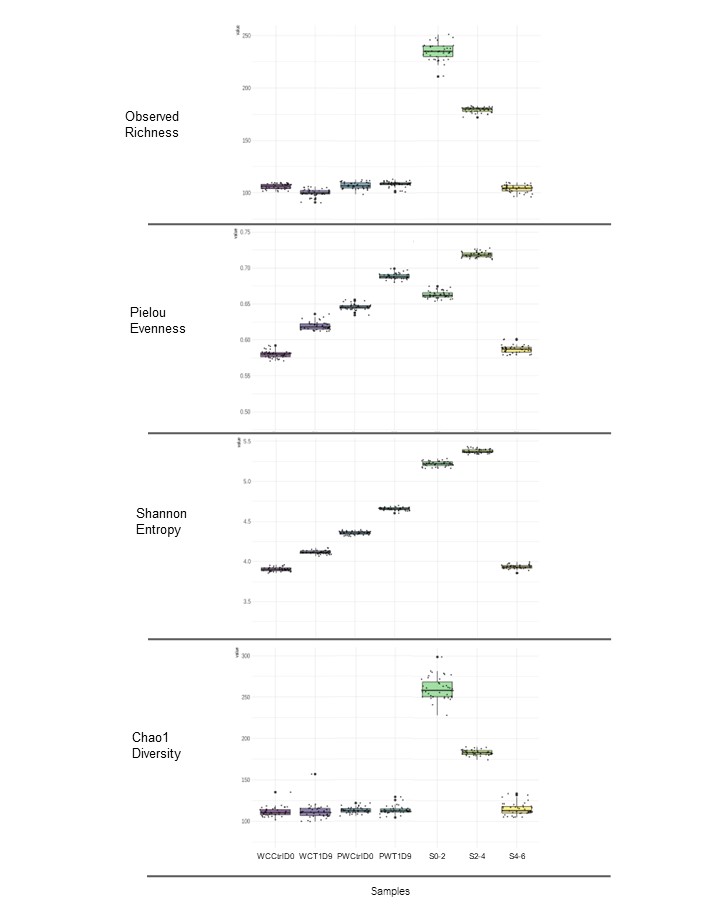

Supplement: Supplementary file 6 [file Image_2.JPEG]
